# Climate Change and Aflatoxin B_1_ in Agriculture Products: A Systematic Review

**DOI:** 10.1002/fsn3.71608

**Published:** 2026-03-02

**Authors:** Behrouz Tajdar‐Oranj, Sima Garshasbi, Nader Akbari, Parisa Shavali‐gilani, Azita Akbari, Parisa Sadighara

**Affiliations:** ^1^ Research Center for Environmental Determinants of Health (RCEDH) Kermanshah University of Medical Sciences Kermanshah Iran; ^2^ Department of Health in Emergencies and Disasters, School of Public Health Tehran University of Medical Sciences Tehran Iran; ^3^ Department of Environmental Health Engineering, Division of Food Safety and Hygiene, School of Public Health Tehran University of Medical Sciences Tehran Iran; ^4^ Student Research Committee Kermanshah University of Medical Sciences Kermanshah Iran

**Keywords:** aflatoxin B_1_, agriculture products, cancer, climate change

## Abstract

The trend toward plant‐based foods is increasing. One of the most important threats to the safety of plant‐based products is aflatoxin B_1_ (AFB1). There is ample evidence that the incidence of food pollution is increasing with climate change. This systematic review analyzed the available evidence of increased exposure to this dangerous toxin through food and its association with climate change. For this purpose, databases were searched with designed keywords. The full text of 63 manuscripts was fully evaluated. The relationship between climate change and increased pollution with this toxic metabolite has been observed. Stressors associated with climate change lower plant defenses against fungi. Controlling climate change will likely be one of the most important strategies in controlling pollution by this mycotoxin. Therefore, all countries are advised to implement the Paris Agreement commitments.

## Graphical abstract

Climate change mainly involves increasing temperatures and concentrations of the greenhouse gas carbon dioxide. These changes increase the expression of genes for mycotoxin synthesis, including aflatoxin B1, in fungi. These stressors also lead to reduced plant defenses against fungal infections. As a result, as fungal and insect contamination of crops increases, farmers are using more pesticides. This leads to reduced food safety. The threats posed by climate change require widespread communication. Governments must urgently seek to reduce greenhouse gas emissions.

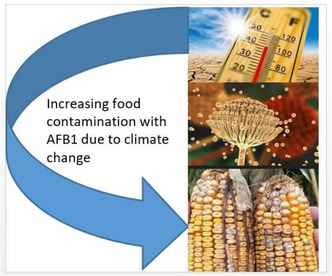

## 1 Introduction

Mycotoxins are secondary metabolites produced by fungi. About 400 types of mycotoxins have been identified. These compounds are stable and can persist throughout the food chain (Xue et al. 2025). Mycotoxins are a major threat to food safety in both developed and developing countries (Chandra 2021). Aflatoxins are a group of mycotoxins produced by Aspergillus species, particularly *Aspergillus flavus* and *Aspergillus parasiticus* (Bianchi et al. 2013). Four types of aflatoxins, G_1_, G_2_, B_1_, and B_2_, are produced by these fungal species. The trend toward consuming plant‐based foods is increasing (Mihalache et al. 2024). These aflatoxins contaminate many plant‐based foods, including grains, oilseeds, cocoa beans, etc., before and after harvest under certain temperature and humidity conditions (Lorán et al. 2022). These fungal species have a high ability to colonize (Nazareth et al. 2019). Therefore, they settle on the plant and cause contamination of food of plant origin. Aflatoxins are heat‐resistant and are not destroyed during food processing or cooking and remain in the final product (Milićević et al. 2019). Aflatoxins B_1_ (AFB_1_) is one of the most dangerous mycotoxins produced by these fungal species (Zhang et al. 2023). According to the classification of the International Agency for Research on Cancer, AFB_1_ is classified as Category 1. Compounds classified in this category are carcinogenic in both humans and laboratory animals (Savić et al. 2020; Roila et al. 2021). AFB_1_ is considered to be the strongest natural carcinogens in the world (Baranyi et al. 2013). Due to its high toxic effects and genetic toxicity, this mycotoxin should be kept to a minimum in food (Gagiu et al. 2025; Mallouki and Luo 2025). The association between this mycotoxin and hepatocellular carcinoma has been confirmed. This mycotoxin is converted in the liver to a carcinogenic metabolite, the epoxide metabolite (Smith et al. 2022). It is converted to the intermediate metabolite 8,9‐epoxide isoform following metabolism by liver enzymes. This metabolite has the potential to interfere with nucleic acids and cause cancer (Gramantieri et al. 2022). According to the regulations of the European Union, the limit of AFB_1_ is 20 μg kg^−1^ for animal feed and 10 μg kg^−1^ for human food (Molnár et al. 2023).

Climate change is one of the threats to food safety (Lee et al. 2018). Two manifestations of climate change are the increase in temperature and the concentration of carbon dioxide gas (Kovač et al. 2022). Climate change has led to an increase in the concentration and frequency of mycotoxin contamination of human and livestock feed (Jaffali et al. 2025). Many studies confirm that the production of aflatoxins depends on the environmental conditions. Temperature and humidity are essential factors for the production of mycotoxins (Kovač et al. 2022; Zhang et al. 2023). Humidity of 22% and temperature between 25°C and 30°C provide the best conditions for the production of mycotoxins (Lanubile et al. 2021; Zhang et al. 2023). These factors are important factors in fungi growth. Therefore, using artificial intelligence and predictive modeling of mycotoxins can be used in mycotoxin management. Input data for the prediction are usually humidity and temperature, type of mycotoxin, soil characteristics and geographical area, and carbon dioxide levels. Using these models, it is possible to estimate the amount of mycotoxins, especially after harvest (Castano‐Duque et al. 2025).

**FIGURE 1:**
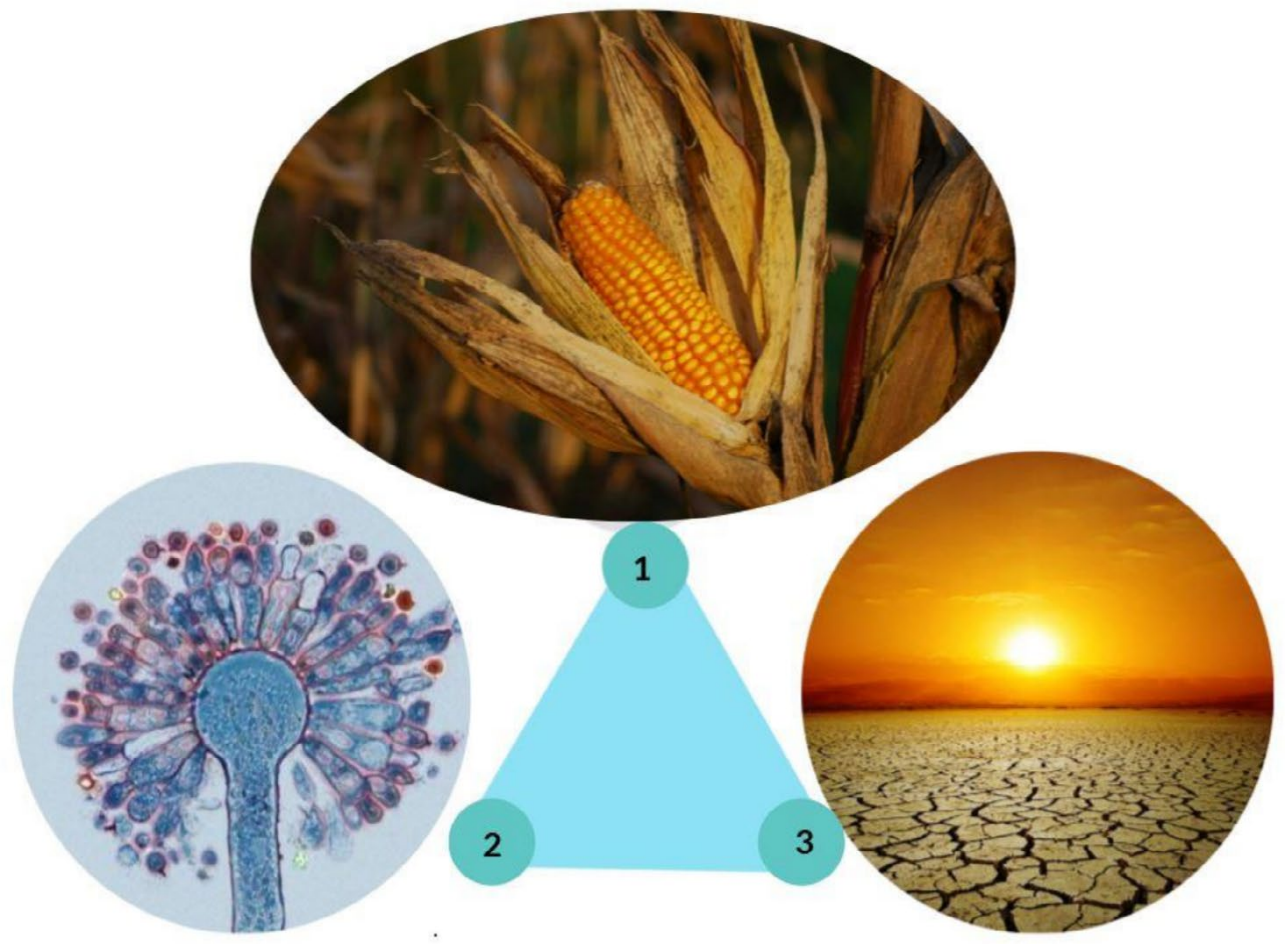
Factors affecting the amount of aflatoxins: (1) Type of plant species, (2) species of fungus producing aflatoxin, (3) environmental conditions.

Aflatoxin‐producing fungus, host (plant) and environmental conditions are related in a triangle (Figure 1). Aflatoxin‐producing fungi cause infection in drought conditions and temperatures of 29°C–35°C. Some plant species like corn have ear silks and are more sensitive to infection. The environmental conditions of different regions are different. Soil type, weather conditions, presence of insects and water retention are all effective in susceptibility to infection with aflatoxins (Barański et al. 2021). Stressors such as heat and drought weaken plant defenses (Mwalugha et al. 2025). If a plant faces a lack of water and there are various pests such as rootworm, the plant becomes sensitive to fungal infections (Kos et al. 2020). It has been observed that climate change leads to an increase in pest species such as Diabrotica. Diabrotica larvae attack the plant roots. In this situation, the plant faces a water and nutrition crisis and becomes more sensitive to fungal infections (Ferrari et al. 2022). In addition, in vitro studies have confirmed that temperature, humidity and carbon dioxide are effective in aflatoxin gene expression (Ferrari et al. 2022).

Tropical regions show a higher abundance of aflatoxin‐producing *A. flavus* with a stronger capacity of these strains to produce aflatoxin, while higher latitude regions have higher abundance of non‐aflatoxigenic and low‐aflatoxigenic *A. flavus*. Mycotoxins caused by *A. flavus* are one of the threats to human health in warm regions (Savić et al. 2020). The occurrence of mycotoxins is increasing due to climate change (Braun et al. 2018; Barany et al. 2021; Khodaei et al. 2021). Their presence is also reported in other regions, which is due to climate change (Melguizo et al. 2023). Factors predisposing to the growth of fungi, including temperature and carbon dioxide, are increasing (Camardo Leggieri et al. 2019). Increased temperature combined with humidity leads to fungal growth (Fumagalli et al. 2021). Global warming has caused the migration of thermophilic micromycetes to cold and temperate regions, so the occurrences of contamination with these mycotoxins have increased in these regions (Voinova et al. 2022). On the other hand, climate change does not only manifest itself in the form of drought and heat. In some cases, climate change has led to the creation of hurricanes, which also provide conditions for the growth of fungi (Savić et al. 2020; Xue et al. 2021). According to the climate change convention, climate change has harmful effects on the economy, human and animal health, and society (Milićević et al. 2019). Climate changes led to the occurrence of aflatoxin‐producing Aspergillus species in areas where they were not present before (Baranyi et al. 2015). Climate change also affects food security in addition to food safety (Leggieri et al. 2021). The increase in temperature and droughts have a negative effect on agriculture. It leads to the reduction of food products (Fanzo et al. 2018).

In this systematic review, the evidence that has been reported in the literature on the occurrence of increased aflatoxin B_1_ contamination by climate change has been collected and discussed.

## 2 Method

### 2.1 Search Process

The 27‐item Prisma checklist was used for this review. Following this checklist will help ensure that we do not miss anything. The search was carried out on December 18, 2025, with these specialized keywords: (“climate change” or “Environmental change” or “Climate disruption” or “Weather change” or “Climate crisis”) and (“aflatoxin b_1_” or “aflatoxin m_1_” or AFB_1_ or AFM_1_). The search was done in three databases: Scopus, PubMed, and Science Direct. To eliminate any bias, this stage of the study was conducted independently by two of the authors, and the results were compared.

### 2.2 Inclusion and Exclusion Criteria

In this study, manuscripts were selected that examined AFB1 in a variety of foods and its relationship with climate change. For this purpose, studies were selected that had data on food contamination with this type of mycotoxin and data from meteorological information. In these studies, the relationship between these two types of data was examined and discussed. Review manuscripts, letters to the editor, and abstracts of congresses, as well as manuscripts that evaluated the toxic effects of aflatoxin, were excluded from the study. Some manuscripts had a routine evaluation and only determined the amount of aflatoxins. Therefore, these manuscripts were excluded from this review. Also, some studies on modeling and predicting the pollution situation in the coming years were excluded from this review. In some studies, contamination was intentional, and the rate of fungal growth was evaluated under laboratory conditions, which was also considered an exclusion criterion.

## 3 Results

### 3.1 The Result of Search

**FIGURE 2:**
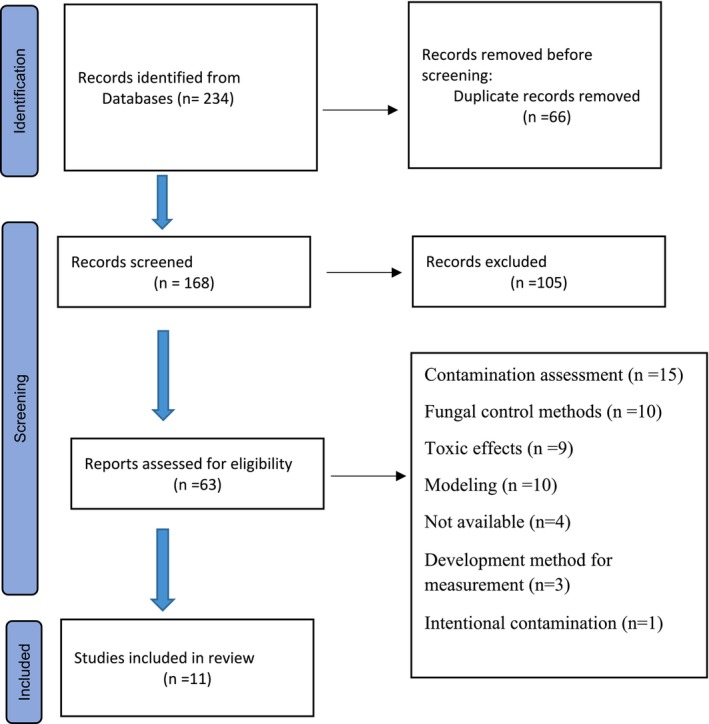
Search process diagram.

The search results in the databases were 234 articles. Selected manuscripts were entered into the Endnote software. Duplicate articles were removed. The titles and abstracts of the remaining 168 articles were carefully read. Articles that met the inclusion criteria were selected. After the initial evaluation, 63 articles were selected for a more complete and detailed evaluation (Figure 2). Of these 63 manuscripts, 11 manuscripts were selected based on the main inclusion criteria, which included the availability of data on mycotoxin levels along with meteorological information.

### 3.2 Data Extraction

Data from the manuscripts were extracted from the manuscripts by two team members. The data in the table included geographical location, year of publication of the study, mycotoxin assessment, and its relationship to meteorological data. Analytical methods were also included (Table 1). The years of research for the selected manuscripts ranged from 2016 to 2025. This confirms that this topic is one of the current topics that researchers have begun to research and study in this regard.

**TABLE 1:** The extracted data according to the protocol.

Study food	Study population	Type of assessment	Type of test	Observed evidence	Geographical region	Author/year
Grape	—	Investigation of *A. flavus* in grape samples during 2019–2021	PCR test	The possibility of increasing the prevalence of *A. flavus* due to climate change	Spain	Melguizo et al. (2023)
Food and feed samples	—	AFB_1_ measurement in food and feed samples during 2017–2019	Measuring aflatoxin with ELISA and investigating the regression of aflatoxin levels with weather conditions.	13.4% of pollution incidents are related to weather conditions	Turkey	Cüce (2020)
Cereal crops	—	AFB_1_ measurement in cereal crops during 2016 and 2017	LC–MS/MS	The increase in the number of samples in 2017 according to the weather conditions	Croatia	Kovač et al. (2022)
Maize samples	—	AFB_1_ measurement in maize in 2014	HPLC	Observing the relationship between the drought index and the amount of AFB_1_	Italy	Leggieri et al. (2020)
Maize samples	—	AFB_1_ measurement in 4 region	HPLC	Confirming the relationship between temperature and pollution level using regression model	Serbia	Janić Hajnal et al. (2017)
Maize samples	—	AFB_1_ measurement in corn samples during 2012–2015	LC–MS/MS	Contamination of 71% of the samples at the same time as the water scarcity crisis	Northern Serbia	Kos et al. (2020)
Maize samples	—	AFB_1_ measurement in 6 region	HPLC	Confirming the link between increased aflatoxins and decreased rainfall	South Africa	Nji et al. (2024)
Maize samples	—	AFB_1_ measurement in maize samples during 2018–2020	HPLC–MS/MS	Higher levels of mycotoxins in 2020 and unfavorable weather conditions this year	France	Bailly et al. (2025)
Agriculture products	—	AFB_1_ measurement in corn samples during 2010–2014	ELISA	Compliance with weather conditions was seen	Italy	Vita et al. (2016)
Pistachio samples	—	Measurement of AFB_1_ in pistachio samples for 5 years under high carbon dioxide exposure conditions	HPLC	Compliance with high carbon dioxide was seen	Saudi Arabia	Baazeem (2021)
Buckwheat grain sample	—	Measurement of aflatoxin B_1_ in wheat grains during the years 2013–2015	ELISA	Direct relationship between aflatoxin B_1_ and drought conditions	Lithuania	Keriene et al. (2018)
Wheat	—	Investigating wheat contamination under drought conditions	ELISA	Observation of a significant association between aflatoxin B_1_ and very dry temperatures	Romania	Gagiu et al. (2025)

Abbreviations: CE, capillary electrophoresis; CE‐MS, capillary electrophoresis coupled to mass spectrometry; LOD, limit of detection; ND, not detected.

**FIGURE 3:**
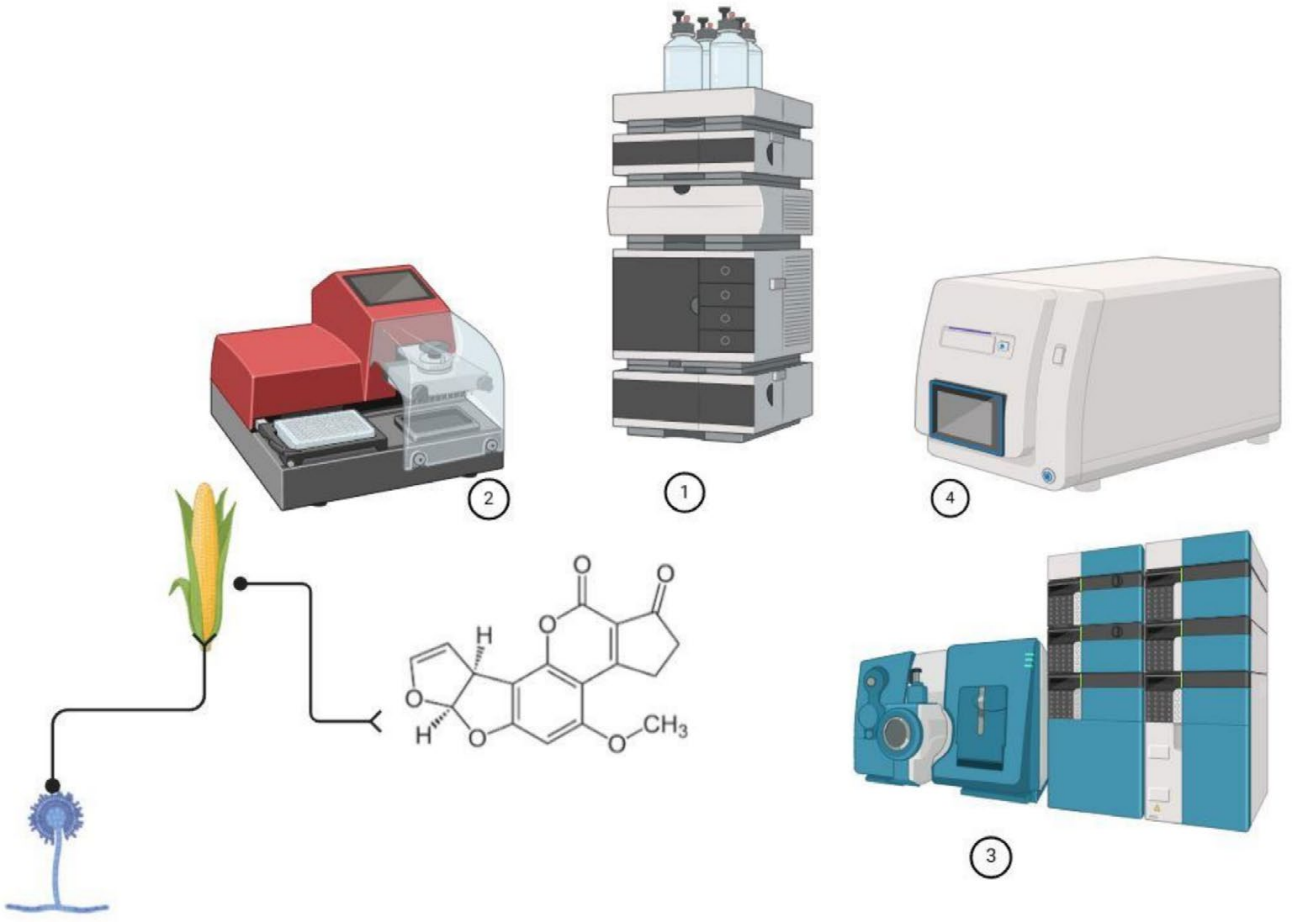
Analytical methods for determining the amount of AFB_1_ in food based on the literature. (1) HPLC, (2) ELISA, (3) LC–MS/MS, (4) PCR.

The most studied product was maize. Maize has a special place among agricultural products. Other agricultural products include pistachios, grapes, and wheat. Different analytical methods have been developed to determine the amount of AFB_1_ in food. Figure 3 shows these methods. The most used method is based on liquid chromatography. These methods have high sensitivity to determine the amount of this mycotoxin (Vaz et al. 2020). HPLC with fluorescence detector (HPLC‐FLD) was the most analytical method among liquid chromatography for measurement. This mycotoxin exhibits strong fluorescence at 425 nm (Zhang and Banerjee 2020). HPLC‐FLD is a common method capable of detecting AFB1 levels at trace amounts in food (Yin et al. 2022). In some studies, ELISA kits were used to determine the amount of AFB1. This method is still used to screen food for mycotoxin contamination (Cao et al. 2024). This method is an inexpensive and rapid method and requires a small sample volume, but in some cases, the accuracy of the method may be affected by sample preparation (Maggira et al. 2022).

**FIGURE 4:**
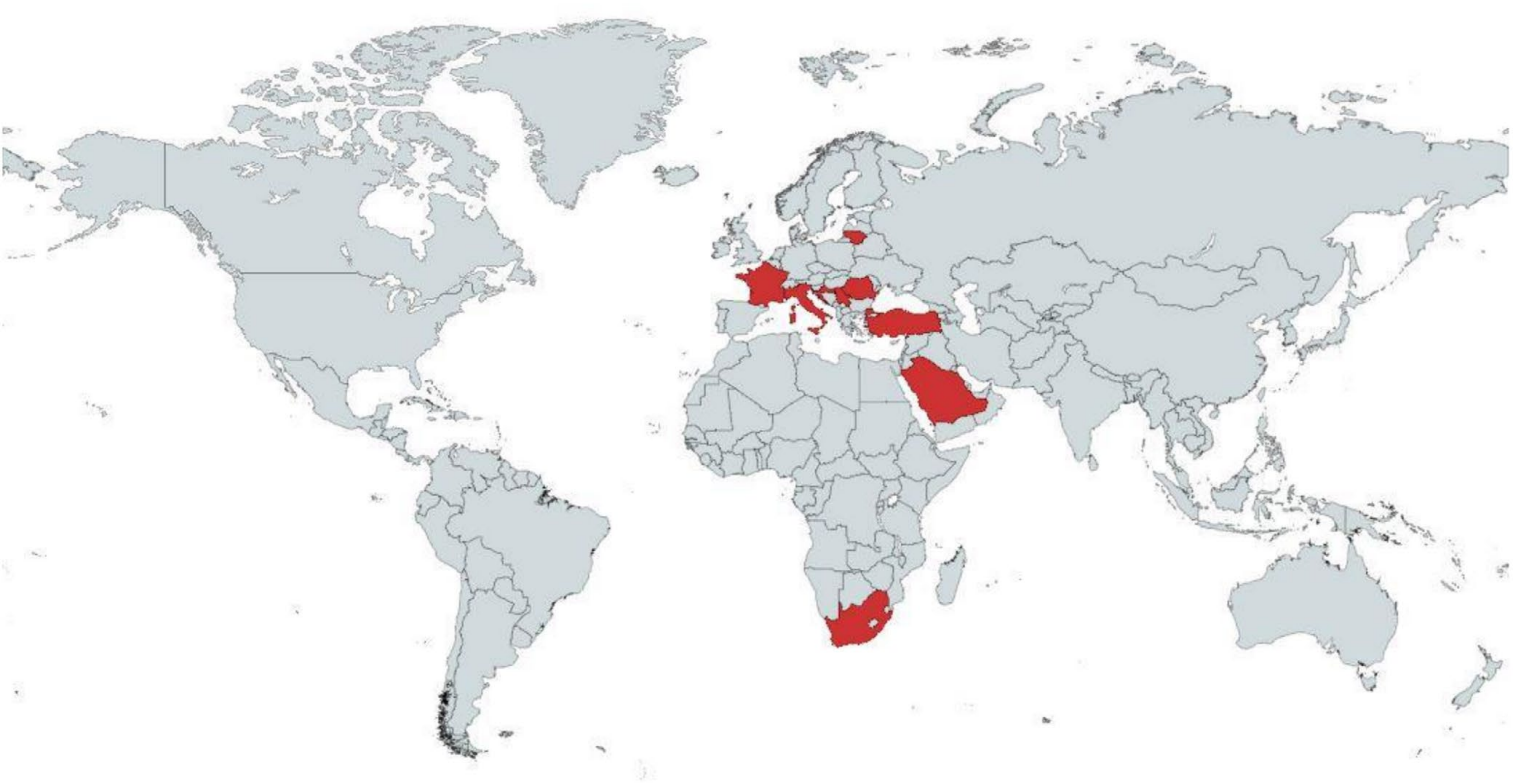
Distribution of studies by geographical area: Red: Location of study.

Figure 4 summarizes the geographical distribution of studies. Most of the studies were conducted in Europe. More limited studies have been conducted on other continents.

## 4 Discussion

One of the biggest threats to human health is climate change (Swinburn et al. 2019; Navas‐Martín et al. 2024). There are currently three major pandemics in the world, including malnutrition, obesity, and climate change (Swinburn et al. 2019). In addition to its effects on human health, climate change can also cause severe economic damage (Abbass et al. 2022). This phenomenon leads to a decrease in water resources and threatens biodiversity (ECA et al. 2020). Furthermore, one of the current crises is providing enough food for the people of the world. Recently, food losses have increased as a result of climate change (Kogan 2023). Plants and grains are the main source of food supply for the people of the world, whose losses have increased in recent years due to climate change (Kogan 2023). In addition to disrupting food security, climate change is also a threat to food safety. Aspergillus species producing AFB_1_ is one of the food safety threats in tropical and subtropical regions (Molnár et al. 2023). *A. flavus* species is a common contaminant in staple items such as rice, peanuts, cotton seed, and corn (Mamo et al. 2021; Tan et al. 2023). This systematic study reviewed all the evidence that has been done on the population or food that shows an increase in exposure to this toxin due to climate change.

In the past, grape vineyards were mainly infected with *Aspergillus niger* and *Aspergillus carbonarius* and to a lesser extent with *Aspergillus steynii* (Melguizo et al. 2023). In the study of Melguizo et al. (2023), it was observed that, due to climate change, temperature increase, drought and rainfall events, and increased carbon dioxide concentration in Spain, contamination with *A. flavus* is seen in grapes. In this study, grape samples were examined for *A. flavus* during the years 2019 to 2021. This fungus was identified in 70% of grape samples (Melguizo et al. 2023). That was a type of fungus that produces the toxic metabolite of AFB_1_.

One of the regions that has been affected by climate change in recent years is Siberia. In this region, an annual rise of 0.07°C is reported every year (Czerniawska and Chlachula 2020). There is evidence of growth of mycotoxin‐producing fungi following warmer autumns in this region (Bogdanova et al. 2021). In a study in North Serbia, the amount of AFB_1_ in corn samples was evaluated between 2012 and 2015. The highest amount of AFB_1_ observed was reported in 2012. Based on meteorological findings, stressful agrometeorological conditions were observed that year. That year, corn fields were faced with water scarcity (Kos et al. 2020). Rootworm and European corn borer pests were present in this area, so the plants became sensitive to infection with fungi. 71% of corn samples were contaminated with AFB_1_ (Kos et al. 2020).

The results of numerous studies indicate an increase in food contamination with aflatoxin B_1_ in Europe and other parts of the world (Janić Hajnal et al. 2017). Due to the increase in temperature in Europe, one of the main topics of food safety is expected: contamination of corn with AFB_1_ (Ferrari et al. 2022). In the Leggieri et al. (2020) study, the relationship between the amount of aflatoxin B_1_ in corn samples and the drought index in some areas of Italy was observed (Leggieri et al. 2020). The aridity index is defined for a time interval of 10 days. The aridity index indicates zero indicates drought in that region. This condition is when the temperature curve is higher than the rainfall (Leggieri et al. 2020). Furthermore, in Italy, during the years 2010–2014, the amount of aflatoxin B_1_ was evaluated in 5 years for climate change. The most common outbreak of infection was in 2012, when the temperature was high this year (Vita et al. 2016).

In the study of Cüce in Turkey, the amount of AFB_1_ was measured in the samples of plant during 3 years from 2017 to 2019. Temperature, humidity, and precipitation were obtained from meteorological data (Cüce 2020). With the help of meteorological data, current and past weather patterns can be analyzed (Haldar et al. 2023). The relationship between the frequency of this mycotoxin and temperature was observed in 2018. By analyzing the regression between the abundance of AFB_1_ and climatic conditions, including temperature, humidity, and rainfall, a 13.4% effect on AFB_1_ contamination was observed (Cüce 2020).

In the study of Kovač et al., AFB_1_ was measured in cereal samples during the years 2016 and 2017. In accordance with this measure, climate changes, including temperature, were also investigated. In 2016, mycotoxin was not detected in all samples, but in 2017, it was detected in the range of 0.4–43.7 μg kg^−1^ (Kovač et al. 2022). The increase in the amount of AFB_1_in cereal samples in 2017 is related to dry annual precipitation amounts in 2017 (Kovač et al. 2022).

In the study of Janić Hajnal et al. (2017), the amount of aflatoxin B_1_ was measured in maize samples in the north, south, west and central of a region in Serbia in 2015 (Janić Hajnal et al. 2017). The lowest amount was observed in the west and the highest amount was observed in the center. A regression model was used and the relationship between the amount of aflatoxin and temperature was measured. A strong correlation between the temperature and the amount of aflatoxins in these four regions was observed (Janić Hajnal et al. 2017). Another study from this country was conducted by Kos et al. They examined the levels of aflatoxin in maize over 4 years. During those 4 years, there have been climatic changes. 2012 was a severe drought, 2013 and 2015 were hot and dry conditions, and 2014 was heavy rainfall. Significant levels of aflatoxin B_1_ contamination were seen in 2012 and 2015, and the authors linked this increase to climate change. The prolonged drought conditions during these 2 years were responsible for the growth of the fungus (Kos et al. 2020). A similar study was conducted in South Africa. A significant number of maize samples were collected from several regions between 2017 and 2021 (Nji et al. 2024). Meteorological data including rainfall and temperature were collected. The lowest aflatoxin contamination was in 2021 and the highest contamination was in 2020. Confirming the link between increased aflatoxins and decreased rainfall (Nji et al. 2024). Another similar study was conducted in France by Bailly and colleagues on maize. For this purpose, maize samples were collected and meteorological information, including temperature and rainfall, was obtained from meteorological stations (Bailly et al. 2025). The study was conducted between 2018 and 2020. The level of contamination in 2020 was higher than the previous 2 years. That year, the weather conditions in this country were not as favorable as in other parts of the world. Maize is an important agricultural crop worldwide, playing a major role in food security and being an important component of the human and livestock diet (Mwalugha et al. 2025).

One of the most widely consumed grains is wheat, which is rich in nutritious compounds, including minerals and vitamins (Deng et al. 2023). In the Keriene et al. (2018) study, the amount of aflatoxin B_1_ in wheat samples was measured during the years 2013–2014. At the same time as weather conditions such as temperature, they also considered the amount of rainfall (Keriene et al. 2018). Aflatoxin B_1_ was not detected in the samples in 2013, but in 2014 and 2015, all samples were positive (Keriene et al. 2018). The authors attributed this result to the drought and high temperatures during those two years. Also, in Romania, the level of contamination of wheat with aflatoxin B_1_ was evaluated under drought conditions (Gagiu et al. 2025). In this study, there was also a relationship between aflatoxin B_1_ and high and dry temperatures, and this relationship was more significant with very dry temperatures.

## 5 Conclusion and Suggested Strategies

During experimental studies, it has been observed that with increasing temperature and concentration of carbon dioxide gas, the expression of the aflatoxin synthesis gene in fungi increases. It was observed that *A. flavus* strains can adapt to high concentrations of carbon dioxide and are able to produce AFB_1_. Subsequently, with the increase in the contamination of agricultural products with mycotoxins, farmers will use more pesticides. The use of chemical antifungals leads to an increase in their residues on food products. This led to the threat of food safety. The use of natural competitors for Aspergillus productive species is one of the suggestions of today's research (Braun et al. 2018). The use of natural competitors such as *Trichoderma harzianum*, *Fusarium verticillioides*, and *Streptomyces roseolus* is recommended (Braun et al. 2018; Caceres et al. 2018; Camardo Leggieri et al. 2019). Furthermore, the use of enzymes and microorganisms is a strategy for the degradation of mycotoxins (Kumar et al. 2018). Policymakers in the agricultural sector must take measures regarding climate change. They should train farmers in this regard and make them more aware.

All countries are required to take measures to reduce carbon monoxide production according to the Paris Agreement in 2015. Based on this Agreement, governments must take measures to reduce greenhouse gas production. Fossil fuels are one of the most important producers of carbon dioxide. Reducing or not using fossil fuels is one of the most important strategies. Using renewable energy sources is one of the suggested strategies. Alternative fuels such as ethanol are also recommended.

## Author Contributions

**Behrouz Tajdar‐Oranj and Sima Garshasbi:** searched in databases. **Nader Akbari, Parisa Shavali‐gilani, and Azita Akbari:** screen the articles. **Parisa Sadighara:** writing – original draft, writing – review and editing.

## Ethics Statement

The authors have nothing to report.

## Consent

The authors have nothing to report.

## Conflicts of Interest

The authors declare no conflicts of interest.

## Data Availability

The datasets generated and analyzed during the current study are available from the corresponding author on reasonable request.
